# Insulin-like growth factor-II overexpression accelerates parthenogenetic stem cell differentiation into cardiomyocytes and improves cardiac function after acute myocardial infarction in mice

**DOI:** 10.1186/s13287-020-1575-4

**Published:** 2020-02-26

**Authors:** Yi Sui, Wei Zhang, Tao Tang, Lili Gao, Ting Cao, Hongbo Zhu, Qinghua You, Bo Yu, Tao Yang

**Affiliations:** 1 Department of Nutrition, The First Affiliated Hospital of Sun Yat-sen University, Guangzhou, 510080 China; 2 Department of Pharmacology, School of Basic Medicine, Hebei University of Chinese Medicine, Shijiazhuang, 050200 China; 3 Department of Obstetrics and Gynaecology, Prince of Wales Hospital, Shatin, New Territories, Hong Kong, China; 4 Center for Medical Research and Innovation, Shanghai Pudong Hospital, Fudan University Pudong Medical Center, Shanghai, 201399 China; 5 Department of Pathology, Shanghai Pudong Hospital, Fudan University Pudong Medical Center, Shanghai, 201399 China; 6 Department of General Surgery, Shanghai Pudong Hospital, Fudan University Pudong Medical Center, Shanghai, 201399 China

**Keywords:** Parthenogenetic stem cells, Insulin-like growth factor-II, Differentiation, Cardiomyocytes, Acute myocardial infarction

## Abstract

**Background:**

Parthenogenetic stem cells (PSCs) are a promising source of regenerated cardiomyocytes; however, their application may be limited without a paternal genome. Insulin-like growth factor-II (IGF-II), a paternally expressed growth hormone, is critical in embryonic differentiation. This study investigated whether forced expression of IGF-II in PSCs can accelerate their differentiation.

**Methods:**

Overexpression and re-knockdown of IGF-II in PSCs were performed to investigate the role of IGF-II in PSC differentiation. The derivatives of PSCs with different IGF-II manipulations were transplanted into infarcted murine hearts to investigate the role of IGF-II in cardiomyocyte differentiation in vivo.

**Results:**

Data showed that the expression of cardiac troponin T and troponin I in IGF-II-PSC outgrowths preceded that of parental PSC outgrowths, suggesting that IGF-II can accelerate PSC differentiation into cardiac lineage. Overexpression of IGF-II accelerated PSC differentiation towards cardiomyocytes while inhibiting PSC proliferation via the IGF-II/IGF1R signaling. Similar to that observed in cardiac marker expression, on differentiation day 24, IGF-II-PSCs showed PCNA and cyclin D2 expression comparable to juvenile mouse cardiomyocytes, showing that IGF-II-PSCs at this stage possess differential and proliferative properties similar to those of juvenile cardiomyocytes. Moreover, the expression pattern of cardiac markers in IGF-II-overexpressing PSC derivatives resembled that of juvenile mouse cardiomyocytes. After transplantation into the infarcted mouse hearts, IGF-II-PSC-derived cardiomyocytes displayed significant characteristics of mature cardiomyocytes, and IGF-II-depletion by shRNA significantly reversed these effects, suggesting the critical role of IGF-II in promoting cardiomyocyte maturation in vivo. Furthermore, IGF-II-overexpressing PSC derivatives reduced collagen deposition and mitochondrial damage in the infarcted areas and improved cardiac function. The re-knockdown of IGF-II could counteract these favorable effects of IGF-II.

**Conclusions:**

These findings suggest that the ectopic expression of IGF-II accelerates PSC differentiation into the cardiac lineage and promotes cardiomyocyte maturation. The underlying process includes the IGF-II/IGF1R signaling, which is involved in the suppressive effect of IGF-II on PSC proliferation. Moreover, transplanting IGF-II-overexpressing PSC derivatives into the infarcted heart could reduce collagen deposition and improve mitochondria biogenesis and measurements of cardiac function, highlighting the importance of IGF-II in the application of PSCs in cardiac regeneration.

## Background

Acute myocardial infarction (MI) is a leading cause of global mortality, accounting for nearly three million deaths worldwide per annum [1]. Although conventional therapies (antiplatelet agents and percutaneous coronary intervention) improve in-hospital mortality, nearly 30% of patients suffer heart failure as a result of continued loss of functional cardiomyocytes [2, 3]. To save a damaged heart, scientists have developed pharmacological and mechanical support therapies to improve blood supply and to salvage the injured myocardia [4–6]. However, an ideal therapeutic strategy would be to replace the infarcted myocardia with functional cardiomyocytes, especially for patients with end-stage heart failure [7].

Human embryonic (ESCs) and induced pluripotent stem cells (iPSCs) are sources of cardiomyocytes. However, their therapeutic applications are limited by ethical concerns, limited post-transplant cell retention, and immunological rejection to allogeneic cells [8, 9]. In a previous study, we presented parthenogenetic stem cells (PSCs) that were haploidentical for major histocompatibility complexes, as an alternative to ESCs and iPSCs in tissue-engineered heart repair with the capacity to differentiate into functional cardiomyocytes [10]. We also generated PSCs that carry cardiac myosin heavy chain-aminoglycoside phosphotransferase (MHC-neo^r^) and enhanced green fluorescence protein (eGFP) to track donor cells during cardiac differentiation and to produce enriched cardiomyocytes by G418 selection [11].

Although murine PSCs exhibit a cardiomyocyte differentiation potential similar to that of ESCs, a 2- to 3-day delay occurs in PSC differentiation, perhaps due to genetic or epigenetic differences between PSCs and ESCs [10]. The PSC genome’s different imprinted expression pattern can be, at least partially, attributed to the parental allele-specific methylation of the differentially methylated regions (DMRs) that control gene transcription [12]. Insulin-like growth factor-II (IGF-II), a member of the IGF family and a paternally expressed growth hormone, plays an important role in embryonic growth and differentiation—binding to IGF1 receptors (IGF1R and IGF2R) and insulin receptors (INSR) [12, 13]. Our previous study found that in murine PSCs, maternally methylated IGF-II DMRs are 100% methylated [10]. This may eliminate the expression of endogenous IGF-II in PSCs. The overexpression of IGF-II in murine ESCs can intensify myogenic differentiation in mice [14]. However, it remains unknown whether eliminating IGF-II expression in PSCs is responsible for their delayed differentiation.

In this study, we transfected a mouse IGF-II-expressing vector into previously generated MHC-neo^r^/pGK-hygro^r^ + MHC-eGFP PSCs [11] and studied the differentiation pattern of the IGF-II-overexpressing clones. Short hairpin RNA (shRNA), which inhibits IGF-II, and IGF1R inhibitor were used respectivelyto investigate possible signaling pathways involved in IGF-II-induced PSC differentiation. To explore the potential therapeutic effects of IGF-II-overexpressing PSCs for MI, we transplanted the cardiomyocyte-enriched derivatives into the infarcted mouse hearts. Our results demonstrate that these genetically modified PSCs may be a novel and promising source of cardiomyocytes for regenerative therapies for acute MI.

## Methods

### PSC and ESC culture

The ESCs and PSCs carrying MHC-eGFP were generated from [C57Bl/6J × DBA/2J] F1 female mice carrying an MHC-eGFP reporter transgene, as previously described [11]. ESCs and PSCs were maintained on freshly prepared STO feeder layers in maintenance medium containing high-glucose Dulbecco’s modified Eagle’s medium (DMEM; Sigma, St. Louis, MO, USA), 15% fetal bovine serum (FBS; Invitrogen, Carlsbad, CA, USA), 100 U/ml leukemia inhibitory factor (LIF), 2 mM l-glutamine, 1% non-essential amino acids (NEAA), 0.1 mM β-mercaptoethanol, 1% sodium pyruvate (Sigma), 25 mM HEPES (Sigma), 100 U/ml penicillin, and 100 μg/ml streptomycin. To facilitate PSC collection for downstream studies, STO cell-conditioned medium (STO-CM) was collected and applied to PSC cultures after dilution with fresh maintenance medium at different ratios (25%, 50%, 75%, and no dilution). ESC line D3 (CRL-1934) was obtained from American Type Culture Collection (ATCC; Manassas, VA, USA).

### Generating IGF-II overexpressing PSCs

The MHC-neo^r^/pGK-hygro^r^ + MHC-eGFP PSCs were generated as previously described [11] and transfected with an IGF-II overexpressing vector (pME 18s-IGF-II) via electroporation at 180 V. The electroporation voltage was optimized as previously described [11]. PSCs were selected, 3 days after transfection, by G418 at 250 μg/ml in optimally diluted STO-CM for 2 weeks, and independent lineages were derived.

### Spontaneous differentiation and G418 selection

Methods for PSC and ESC differentiation and cardiomyocyte enrichment have previously been described in detail (Fig. 2a) [11]. For differentiation induction, PSCs were cultured in an induction medium. Hanging drop culture [12] was conducted for 2 days (days 1–2), followed by suspension culture for 3 days (days 3–5) and adherent culture for 5 days (days 6–10). For cardiomyocyte enrichment, G418 selection (250 μg/ml) began at day 10. On day 13, the adherent colonies were incubated with 2 mL digestion solution containing IMDM supplemented with 0.2% collagenase type II (Sigma) and 60 U/ml DNase I (Sigma) at 37 °C for 1 h. The resulting cell clumps were collected and resuspended in 0.05% trypsin (Invitrogen) for further digestion for 5 min. The dissociated cells were then pelleted and replated into the G418-supplemented induction medium in 0.1% gelatin-coated dishes for additional enrichment for 12 days (days 13–24). While temporally detecting eGFP^+^-cell purity, data were acquired in 4 individual experiments, conducted in 12 replicates.

### A murine model of acute MI and cell transplant

Animal care and all experimental procedures were performed in accordance with the Guide for the Care and Use of Laboratory Animals published by the US National Institutes of Health (publication No. 85–23, revised 1996). The handling of the mice and all experimental procedures were approved by the Institutional Animal Care and Use Committee of Fudan University. Animals were anesthetized with inhalation of 3% halothane and maintained on 1.5% halothane in 70% nitrous oxide and 30% oxygen. MI was induced via ligation of the left anterior descending artery with a 10-0 prolene suture (Ethicon, Johnson & Johnson Inc., New Brunswick, NJ, USA) via a left thoracotomy at the fourth intercostal space. Infarction was considered successful following the appearance of pale discoloration and an ST elevation on electrocardiograms. PSC- and ESC-derived cardiomyocyte cultures were digested with trypsin, washed three times with PBS, and resuspended in a pro-survival cocktail consisting of 50% (v/v) growth factor-reduced Matrigel (BD Biosciences, Bedford, MA, USA), 100 mM ZVAD (benzyloxycarbonyl-Val-Ala-Asp(O-methyl)-fluoromethyl ketone; Calbiochem, San Diego, CA, USA), 50 nM Bcl-XL BH4 (cell-permeant TAT peptide, Calbiochem), 200 nM cyclosporine A (Novartis, Basel, Switzerland), 100 ng/mL IGF-1 (Sigma), and 50 mM pinacidil (Sigma) [13]. 1 × 10^5^ cells in 10 μl pro-survival cocktail solution were injected into two sites adjacent to the infarcted tissue of anesthetized, intubated [C57Bl/6J × DBA/2J] F1 female siblings (12 ± 1 weeks old; 29 ± 3 g; *n* = 20) with a 30-gauge tuberculin syringe at 1 week after infarction. After extubation and evacuation of the pneumothorax, the mice were situated at 37 °C and monitored in micro-isolator cages (one per cage) until they had recovered from surgery. To collect the heart samples, all mice were sacrificed by CO_2_ asphyxiation.

### Semi-quantitative PCR and quantitative real-time PCR (qRT-PCR)

Total DNA and RNA were extracted using the TRIzol Reagent (Invitrogen), according to the manufacturer’s instructions. This was followed by cDNA synthesis via reverse transcription reaction (RNeasy extraction mini kit; Qiagen, Hilden, Germany). Semi-quantitative PCR was performed, and the PCR products were separated on ethidium bromide-stained agarose gels. Images were collected using Image Lab (Bio-Rad, Hercules, CA, USA). The qRT-PCR was performed in an ABI Prism 7000 Sequence Detector (Applied Biosystems, Foster City, CA, USA) using SYBR Green PCR Master Mix reagent following the manufacturer’s instructions. Gel electrophoresis and melting curve analyses were performed to confirm PCR product size and the absence of non-specific bands. The primer sequences were as follows: *β-actin*: (forward) *CGA GGC CCA GAG CAA GAG*, (reverse) *CGT CCC AGT TGG TAA CAA TGC*; *Nanog*: (forward) *TGC TAC TGA GAT GCT CTG CAC A*, (reverse) *TGC CTT GAA GAG GCA GGT CT*; *Oct-4*: (forward) *GCC CCA ATG CCG TGA AG*, (reverse) *CAG CAG CTT GGC AAA CTG TTC*; *Rex-1*: (forward) *GGC CAG TCC AGA ATA CCA GA*, (reverse) *GAA CTC GCT TCC AGA ACC TG*; *FoxD3*: (forward) *GTC CGC TGG GAA TAA CTT TCC GTA*, (reverse) *ATG TAC AAA GAA TGT CCC TCC CAC CC*; *α-MHC*: (forward) *GCT GAC AGA TCG GGA GAA TCA G*, (reverse) *CCC CTA TGG CTG CAA TGC*; *β-MHC*: (forward) *TCC TCA CAT CTT CTC CAT CTC TGA*, (reverse) *GCA AAA TAT TGG ATG ACC CTC TTA G*; *cTnT*: (forward) *CAG AGG AGG CCA ACG TAG AAG*, (reverse) *CTC CAT CGG GGA TCT TGG GT*; *IGF-II*: (forward) *TGT GCT GCA TCG CTG CTT AC*, (reverse) *AAA CTG AAG CGT GTC AAC AAG CT*; *IGF type 1 receptor* (*IGF1R*): (forward) *CAT GCA GGA GTG TCC CTC*, (reverse) *TGA GCA GAA GTC ACC GAA TC*; *IGF type 2 receptor* (*IGF2R*): (forward) *TTA CAC ATG GGA AGC TGT TGA CT*, (reverse) *CGG CAG TTC TCT GTC TTT AGG TC*; *insulin receptor* (*INSR*): (forward) *GCT ACA TCT GAT TCG AGG AGA G*, (reverse) *TGA GTG ATG GTG AGG TTG TG*. The expression level of each target gene was normalized to the β-actin level using the comparative C_T_ method. Data were presented as fold change in expression, relative to controls.

### Western blot analysis

Cells were lysed with lysis buffer. Protein concentration was determined using a BCA protein assay reagent (Cat# 71285; Millipore, Billerica, MA, USA). Twenty micrograms of protein was separated by 12% SDS-PAGE gel, transferred to polyvinylidene fluoride membranes (Millipore), blocked for 1.5 h with Tris-buffered saline containing Tween 20 (TBST) with 1% bovine serum albumin at room temperature, and incubated overnight with primary β-actin antibodies (1:2000, rabbit mAb, cat# MABT523, clone RM112; Millipore), cardiac troponin T (cTnT; 1:1000, mouse mAb, cat# MABT368, clone 9C2.1; Millipore), cardiac troponin I (cTnI; 1:1000, rabbit pAb, cat# 4002S; Cell Signaling Technology, Beverly, MA, USA), proliferating cell nuclear antigen (PCNA; 1:1000, rabbit mAb, cat# ab92552, clone EPR3821; Abcam, Cambridge, MA, USA), cyclin D2 (1:500, rabbit pAb, cat# SAB4301582; Sigma), IGF-II (1:1000, rabbit pAb, cat# ab170304; Abcam), IGF1R (1:1000, mouse mAb, cat# 05-1106, clone 1-2; Millipore), phosphorylated IGF1R (1:500, rabbit pAb, cat# ABE332; Millipore), IGF2R (1:1000, rabbit mAb, cat# 14364S, clone D3V8C; Cell Signaling Technology), INSR (1:1000, mouse mAb, cat# MABS65, clone CT-3; Sigma), or phosphorylated INSR (p-INSR; 1:500, rabbit pAb, cat# SAB4504613; Sigma) at 4 °C. The membranes were washed three times with TBST, incubated with horseradish peroxidase-conjugated goat anti-rabbit or anti-mouse IgG or IgM (1:2000; Millipore) for 1 h at room temperature, and washed with TBST. The chemiluminescence signal was detected using ECL (GE Healthcare Bio-Sciences, Pittsburgh, PA, USA) and developed on X-ray films. β-actin was used as an internal control.

### Immunofluorescence staining

Tissue sections were subjected to immunostaining for α-actinin (1:100, mouse mAb, clone AT6/172, cat# 05-384; Millipore) following a standard protocol. Sections were incubated with the aforementioned antibody, followed by further incubation with tetramethylrhodamine isothiocyanate (TRITC)-conjugated goat anti-mouse IgG (1:100; Millipore). Rinsed sections were counter-stained with 10 μg/ml Hoechst 33342 (Sigma). For cyclin D2 (1:100, rabbit pAb, cat# ab230883; Abcam) staining in cells, the biotinylated goat anti-rabbit IgG (1:100; Vector, Burlingame, CA, USA) was applied to cells incubated with the anti-cyclin D2. After rinsing, streptavidin-HRP (Vector) was added and immunoreactivity was visualized using diaminobenzidine (Vector). An inverted microscope (IX83; Olympus, Tokyo, Japan) was used for visualization.

### *IGF-II* shRNA inhibition

The *IGF-II*-specific shRNA was designed as previously described [15]. The two complementary shRNA oligonucleotides were 5′-ACC GAC GCC TGC GCA GAG GCC TTT CAA GAG AAG GCC TCT GCG CAG GCG TCT TTT TC-3′ and 5′-TCG AGA AAA AGA CGC CTG CGC AGA GGC CTT CTC TTG AAA GGC CTC TGC GCA GGC GT-3′. The oligos for scrambled RNAi were 5′-ACC GAT ATC CGG TAC CGA AGG TTT CAA GAG AAC CTT CGG TAC CGG ATA TCT TTT TC-3′ and 5′-TCG AGA AAA AGA TAT CCG GTA CCG AAG GTT CTC TTG AAA CCT TCG GTA CCG GAT AT-3′. The double-stranded oligonucleotides were ligated into RNAi-Ready pSIREN-DNR-DsRed-Express Vector (Clontech, Mountain view, CA, USA) and then inserted into Adeno-XLP CMV Vector (Clontech) to construct the recombinant pLP-Adeno-X *IGF-II* shRNA. Oligos were annealed and then cloned downstream of a human U6 promoter in a previously described vector [16]. An expression cassette containing the U6 promoter with the RNAi sequence was then excised and subcloned between the *XhoI* and *XbaI* sites in pAdTrack. Recombinant adenoviruses were generated and amplified in 293 cells (human embryonic kidney cell line; Invitrogen) cultured in DMEM supplemented with 10% FBS (Sigma), as previously reported [17].

### Heart function exams

Transthoracic echocardiography was conducted 4 weeks post-transplant using an echocardiographic system (Vevo 770, VisualSonics Inc., Toronto, Canada) equipped with linear array probes (40 MHz). Mice were anesthetized with 1.5% isoflurane until the heart rate had stabilized at 400–500 beats per minute. The hearts were imaged in a two-dimensional short-axis mode. Left ventricular internal diameter and wall thickness during diastole and systole, ejection fraction, and fractional shortening were calculated with the Vevo Analysis software (version 2.2.3).

### Tissue and serum analysis

After the evaluation of heart function, the mice were euthanized, and the hearts arrested in diastole by intravenous cadmium chloride injection. The hearts were then excised and fixed in 4% paraformaldehyde. Ten 4-μm-thick transverse sections from each heart were sampled from the midpoint between the apex and base, followed by Masson’s trichrome staining. This was done to visualize the myocardial architecture and to quantify the extent of fibrosis, i.e., the percentage of the collagen area in cyan or green that had been automatically detected by Image-Pro Plus (Media Cybernetics, Rockville, MD, USA). IGF-II levels in serum (Quantikine ELISA Kit, cat# MG200, R&D Systems, Minneapolis, MN, USA), heart tissue (DuoSet ELISA, cat# DY792, R&D Systems), vascular endothelial growth factor (VEGF) levels in serum (cat# ab100751; Abcam), and heart tissue (cat# ab209882; Abcam) were determined with ELISA kits following the manufacturer’s instructions.

### Transmission electron microscopy (TEM) analysis

A 1-mm^3^ target tissue surrounding the injection site was prefixed with 2.5% glutaraldehyde for 2 h and post-fixed with 1% osmic acid for an additional 2 h at 4 °C. This was followed by gradient dehydration in 30%, 50%, and 70% ethanol (10 min each); 80%, 90%, and 95% acetone (10 min each); and 100% acetone (10 min twice). Tissues were then embedded in the resin and stained with lead citrate. The stained sections were observed and imaged under a transmission electron microscope (H-7500, Hitachi, Tokyo, Japan). The mitochondrial volume density was then quantified, as previously described.

### Statistical analysis

Data were expressed as mean ± standard error. The analysis was performed using a Student’s *t* test or one-way ANOVA with SPSS 16.0 (SPSS Inc., IL, USA). *P* < 0.05 was considered statistically significant.

## Results

### Generation of IGF-II-overexpressing PSCs and directed cardiomyocyte differentiation

**Fig. 1:**
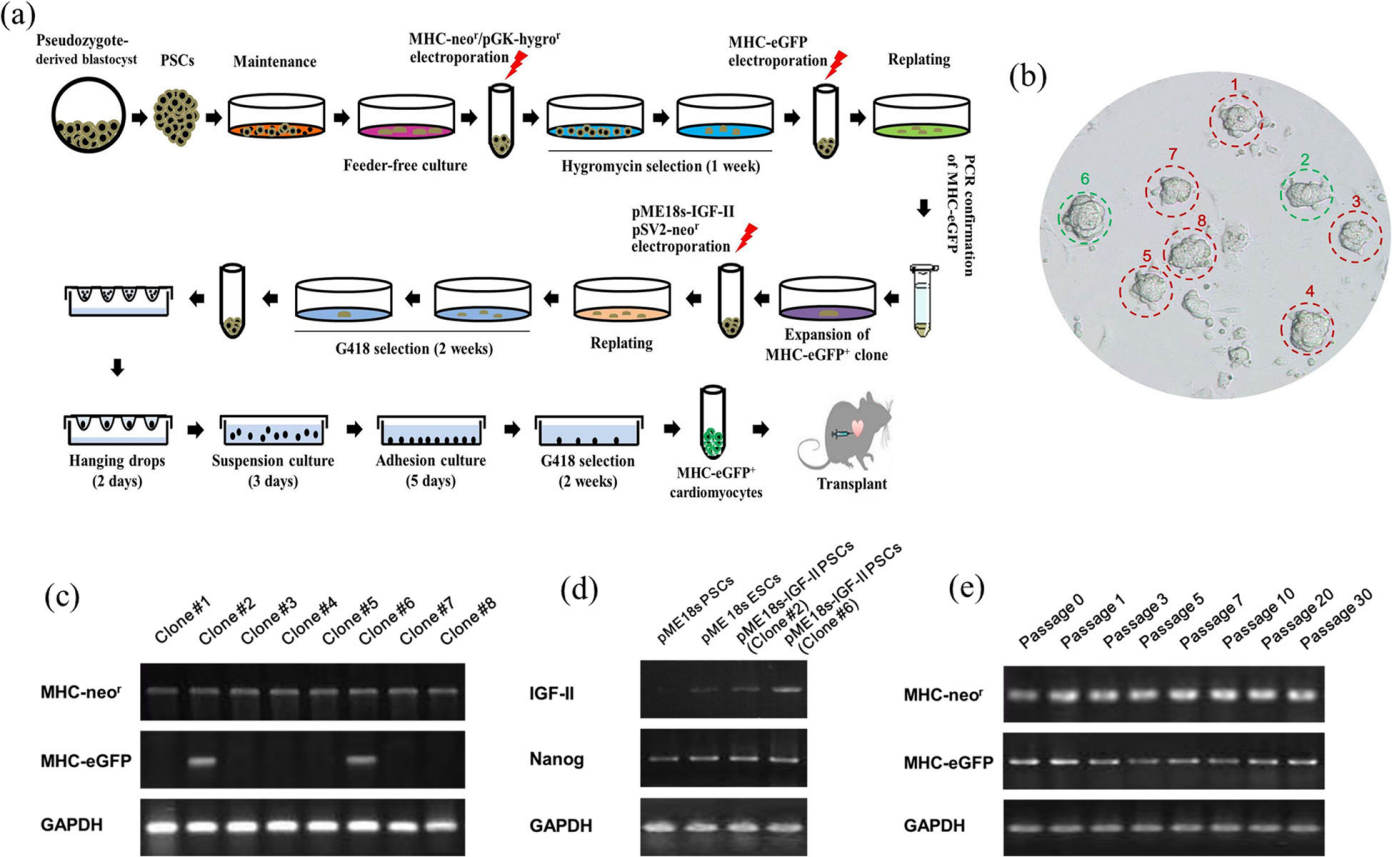
Parthenogenetic stem cell (PCS) generation coexpressing insulin-like growth factor-II (IGF-II) and enhanced green fluorescent protein (eGFP)-tagged myosin heavy chain (MHC). **a** Schematic diagram of parthenogenetic stem cell (PCS) transfection, selection, differentiation, cardiomyocyte enrichment, and engraftment. Undifferentiated embryonic stem cell (ESC) line D3 and mouse-derived ESCs were used as controls and underwent the same procedures. **b** Mouse-derived PCSs were sequentially and stably transfected with MHC-neor/pGK-hygror and MHC-eGFP via electroporation at 180 V. Eight PSC clones (#1–8) were generated. **c** Semi-quantitative PCR was conducted to determine MHC-neo^r^ and MHC-eGFP expression in the genome. **d** Clones #2 and #6 containing MHC-neo^r^ and MHC-eGFP were stably transfected with pME 18 s-IGF-II via electroporation. Semi-quantitative PCR determined IGF-II and Nanog expression in the genome. **e** MHC-neo^r^(/pGK-hygror) and MHC-eGFP expression were determined in multiple passages of clone #6 derivatives

After sequentially transfecting mouse-derived PSCs with multiple vectors (Fig. 1a) [11], we identified a G418-resistant clone (#6) containing MHC-neo^r^/pGK-hygro^r^, MHC-eGFP, and pME 18s-IGF-II plasmids (Fig. 1b–d). PCR analysis showed stable MHC-eGFP expression in clone #6 through consecutive passages (Fig. 1e).

**Fig. 2:**
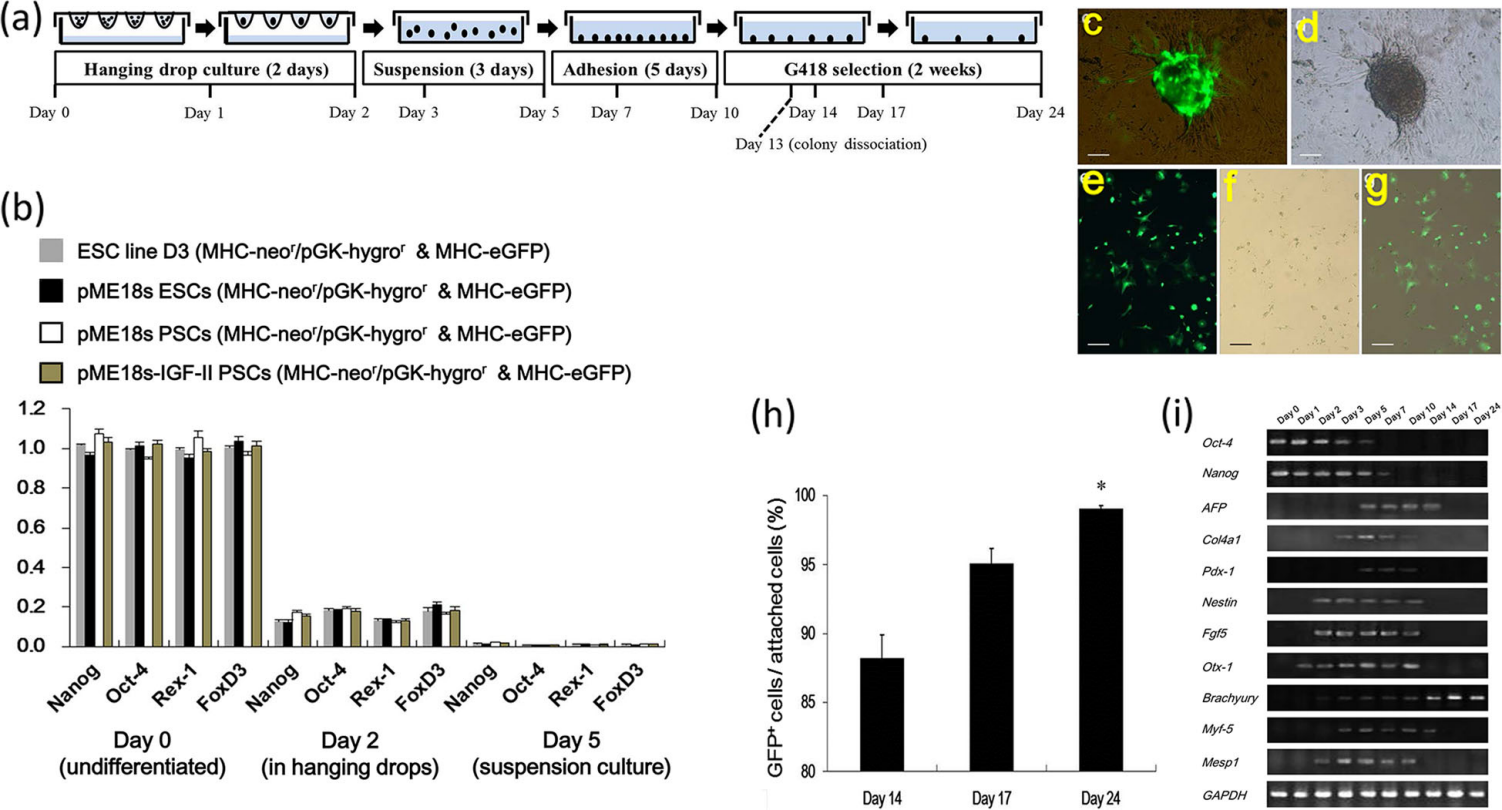
PSC differentiation and cardiomyocyte enrichment. **a** PSC clone #6 (passage 3) underwent directed differentiation and cardiomyocyte enrichment, as indicated. ESC line D3 and mouse-derived ESCs were used as controls and subjected to the same procedures. **b** Pluripotency marker MRNA levels were determined with quantitative real-time PCR (qRT-PCR at days 0, 2, and 5 after differentiation). Data were normalized to the mRNA level of each gene in ESC line D3. Data are expressed as the mean ± standard deviation (SD). *n* = 8. **c** eGFP expression in PSC-derived cardiomyocytes was detected by fluorescence microscopy at day 13 before enzymatic dissociation. **d** Bright-field image of **c**. **e** Cardiomyogenic colonies in **c** were dissociated into single cells at day 13 post differentiation, followed by culture in G418-containing induction medium. At day 24 post differentiation, enriched eGFP^+^ cardiomyocytes were derived. **f** A bright-field image of **e**. **g** A merged image of **e** and **f**. Scale bar: 30 μm for **c** and **d**; 10 μm for **e**–**g**. **h** The percentage of eGFP^+^ cardiomyocytes under G418 pressure at different time points as indicated. Data are expressed as the mean ± SD (*n* = 8). **i** RT-PCR analysis for pluripotent (Oct-4 and Nanog), endodermal (AFP, Cal4a1, and Pdx-1), ectodermal (Nestin, Fgf5, and Otx-1), and mesodermal (Brachyury, Myf-5, and Mesp1) markers at different time points after differentiation

Clone #6 (pME18s-IGF-II PSCs) and the control clones (empty pME18s vector-transfected D3 cell lines, ESCs, and PSCs) were then subjected to spontaneous differentiation and cardiomyocyte enrichment (Fig. 2a). During differentiation, all clones exhibited a progressive decline in the pluripotency markers’ mRNA levels (Oct-4, Nanog, Rex-1, and FoxD3) (Fig. 2b), indicating that the IGF-II-overexpressing PSCs retained differentiation ability comparable to the control clones. The increasing number of eGFP^+^ cells during G418 selection indicated the progressive enrichment of PSC-derived cardiomyocytes (Fig. 2c–h). By monitoring the gene expression during PSC differentiation and cardiomyocyte enrichment (Fig. 2i), we observed that the pluripotency markers (Oct-4, Nanog) decreased after the initiation of differentiation and became invisible on day 7 or 10, whereas the germ layer markers appeared upon differentiation. Endodermal (AFP, Cal4a1, and Pdx-1) and ectodermal (Nestin, Fgf5, and Otx-1) markers disappeared upon G418 selection, whereas mesodermal marker Brachyury remained elevated during selection (days 10–24). This indicated an enriched mesodermal population and cardiomyocyte lineage commitment.

### IGF-II overexpression promotes PSC cardiomyogenic differentiation and maturation in vitro at the expense of proliferation

**Fig. 3:**
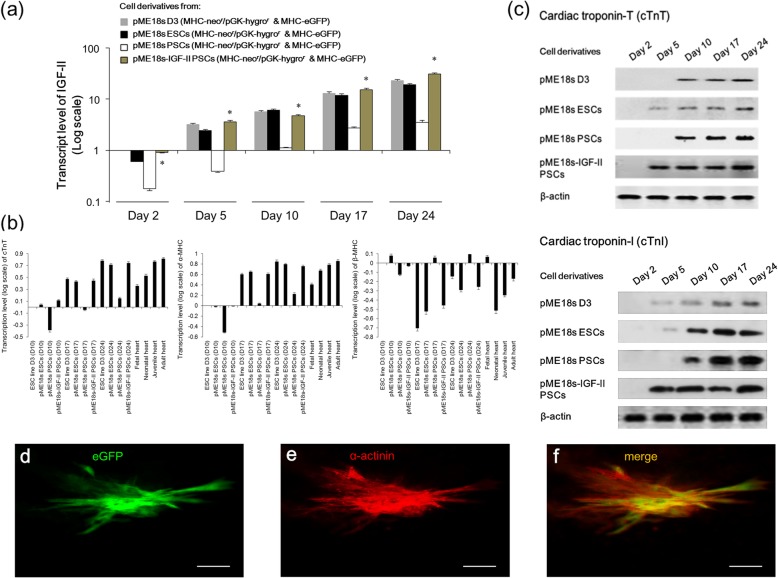
IGF-II overexpression promotes PSC cardiomyogenic differentiation in vitro. **a** qRT-PCR analysis of IGF-II mRNA levels in 1 × 10^6^ PSC- or ESC-derived cells at different time points after differentiation. Data are presented as the log fold-change in mRNA expression normalized to that in untransfected ESC line D3 cells at day 2 after differentiation. Data are expressed as mean ± SD. *p < 0.05 vs. pME 18s PSCs. *n* = 8. **b** qRT-PCR analysis (*n* = 8) of cardiomyocyte-specific markers (cTnT, α-MHC, and β-MHC) in 1 × 10^6^ PSC- or ESC-derived cells at different time points after differentiation. The mRNA levels for cTnT, α-MHC, and β-MHC were also detected in 1 × 10^6^ cardiomyocytes isolated from mouse fetal (14 days post coitus), neonatal (1-day post-birth), juvenile (2 weeks post-birth), and adult (10 weeks post-birth) hearts, respectively. Data are presented as the log fold-change in mRNA expression normalized to that in ESC line D3 cells at day 10 after differentiation. *p < 0.05 vs. pME 18s ESC- or pME 18s PSC-derived cells at day 10 (D10); #*p* < 0.05 vs. pME 18s ESC- or pME 18s PSC-derived cells at day 17 (D17); ∆*p* < 0.05 vs. pME 18s ESC- or pME 18s PSC-derived cells at day 24 (D24), but *p* > 0.05 vs. juvenile cardiomyocytes. **c** Western blot analysis was performed to detect the cTnT and cTnI protein expression in cells derived from pME 18s-IGF-II PSCs at different time points after differentiation. β-actin was used as an internal control. **d**–**f** To demonstrate the maturation of cardiomyocytes derived at day 24, immunofluorescence study of α-actinin was conducted. **d** The eGFP fluorescence. **e** Immunostaining of α-actinin. **f** Merge of **d** and **e**. Scale bar, 25 μm

To examine the role of IGF-II in PSC cardiomyogenic differentiation, we detected the temporal expression of IGF-II and cardiac markers (cTnT, cTnI, α-MHC, and β-MHC) in the outgrowths of different clones. The qRT-PCR results confirmed the negligible expression of endogenous IGF-II in PSCs compared with D3 cells and ESCs, as well as the forced expression of exogenous IGF-II in IGF-II-PSCs (Fig. 3a). Although all clone derivatives exhibited a time-dependent increase in cardiac marker transcripts during differentiation on days 10–24 (Fig. 3b), the empty vector-transfected PSC outgrowths expressed significantly less cardiac markers than the other derivatives. This was observed throughout the differentiation process. In contrast, IGF-II-PSC outgrowths had increased mRNA levels for these markers, compared with the empty vector-transfected PSC outgrowths throughout differentiation. This suggests that IGF-II overexpression promotes PSC differentiation into cardiomyocytes. In addition, the cardiac marker expression trend (days 10–24) for each derivative was similar to that of the cardiomyocytes from fetal (14 days post-coitus), neonatal (1 day post-birth), juvenile (2 weeks post-birth), and adult (10 weeks post-birth) mice. At differentiation day 24, IGF-II-PSC derivatives expressed amounts of cTnT, α-MHC, and β-MHC transcripts comparable to those of juvenile mouse cardiomyocytes. This suggests that at this stage, IGF-II-PSCs may be an ideal cardiomyocyte source for robust cardiac regeneration. Furthermore, Western blot analysis showed that the expression of cTnT and cTnI proteins in IGF-II-PSC outgrowths preceded that of parental PSC outgrowths (day 5 vs. day 10) (Fig. 3c). This suggests that IGF-II can accelerate PSC differentiation into cardiomyocytes. To attest to the maturation of cardiomyocytes derived at day 24, immunofluorescence study on the specific marker of cardiac sarcomere, α-actinin, was conducted, which further confirmed the maturation phenotype of the derived cardiomyocytes (Fig. 3d–f).

**Fig. 4:**
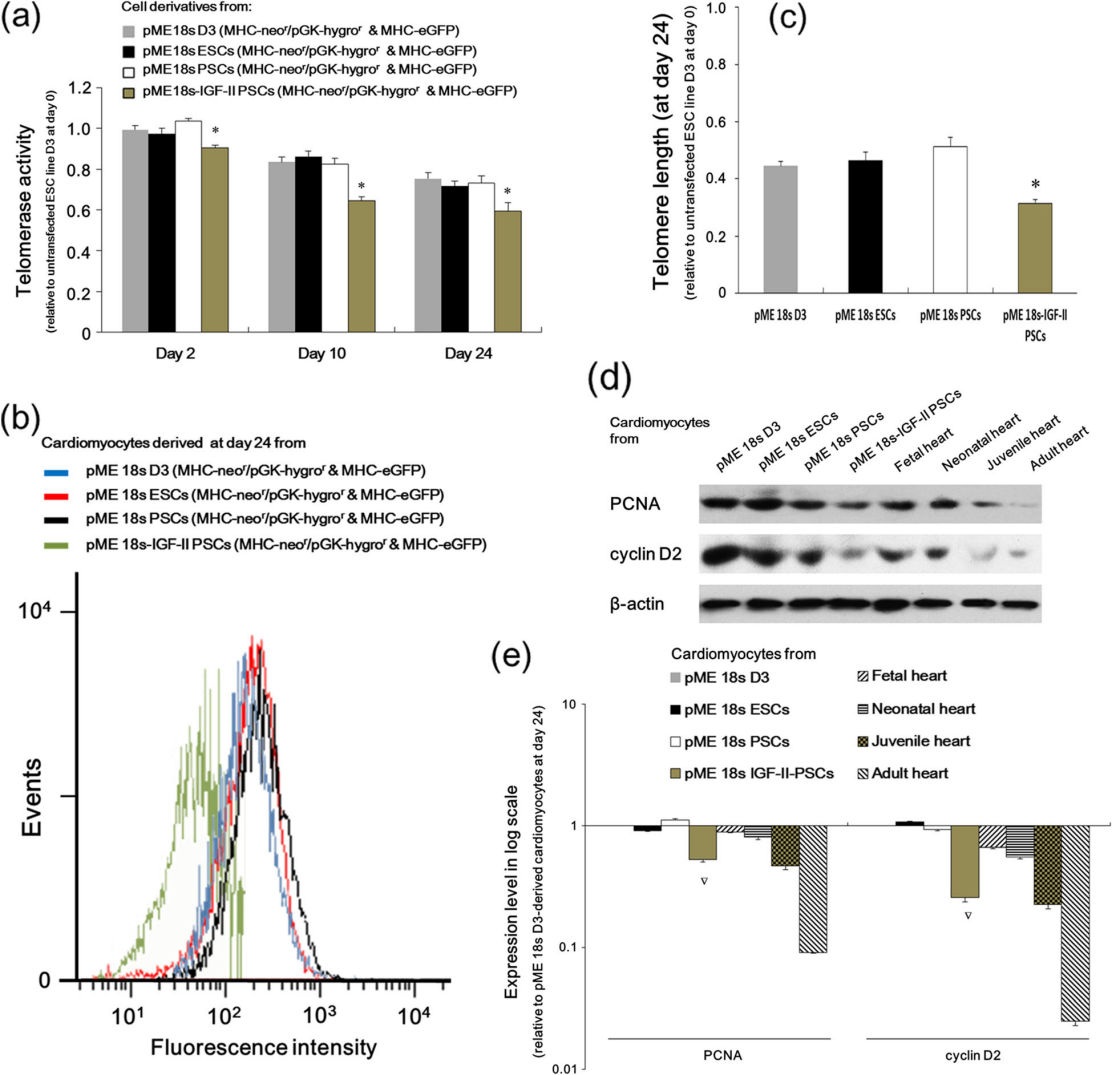
IGF-II overexpression affected telomerase activity, telomere length, and cell cycle regulator expression during PSC differentiation. **a**–**c** The telomerase activity (**a**) at differentiation days 2, 10, and 24 and telomere length at differentiation day 24. (**b**, **c**) was detected in cardiomyocytes derived from various clones. Data in **a** and **c** are presented as the fold change relative to the untransfected ESC line D3 at day 0. *p < 0.05 vs. all other groups. **d** Cell derivatives from various clones were collected at differentiation day 24. Fetal, neonatal, juvenile, and adult hearts were enzymatically digested, followed by cardiomyocyte purification. 1 × 10^5^ cardiomyocytes originated from different sources were analyzed by Western blot to detect protein expression of cell cycle regulators, proliferating cell nuclear antigen and cyclin D2. **e** Quantification of **d**. The expression level of each band was normalized to that of β-actin. Data are presented as a logarithmic scale of the fold change relative to the expression in the pME 18s D3 group. ∆*p* < 0.05 vs. pME 18s PSCs or ESCs, but *p* > 0.05 vs. juvenile cardiomyocytes

Considering the inverse association between cell proliferation and differentiation [18], we detected cell proliferation-associated telomerase activity and telomere length [19] for each differentiation derivative. As shown in Fig. 4a–c, IGF-II-PSC derivatives exhibited significantly less telomerase activity and telomere length than other derivatives during differentiation. This suggests that IGF-II promotes PSC differentiation, possibly at the expense of its proliferation. Results were consistent in the expression of proliferating cell nuclear antigen (PCNA) and cyclin D2 (Fig. 4d and e), two important proteins in cell cycle regulation [20]. Similar to that observed in cardiac marker expression, on differentiation day 24, IGF-II-PSCs showed PCNA and cyclin D2 protein expression comparable to juvenile mouse cardiomyocytes. This suggests that IGF-II-PSCs at this stage possess differential and proliferative properties similar to those of juvenile cardiomyocytes.

**Fig. 5:**
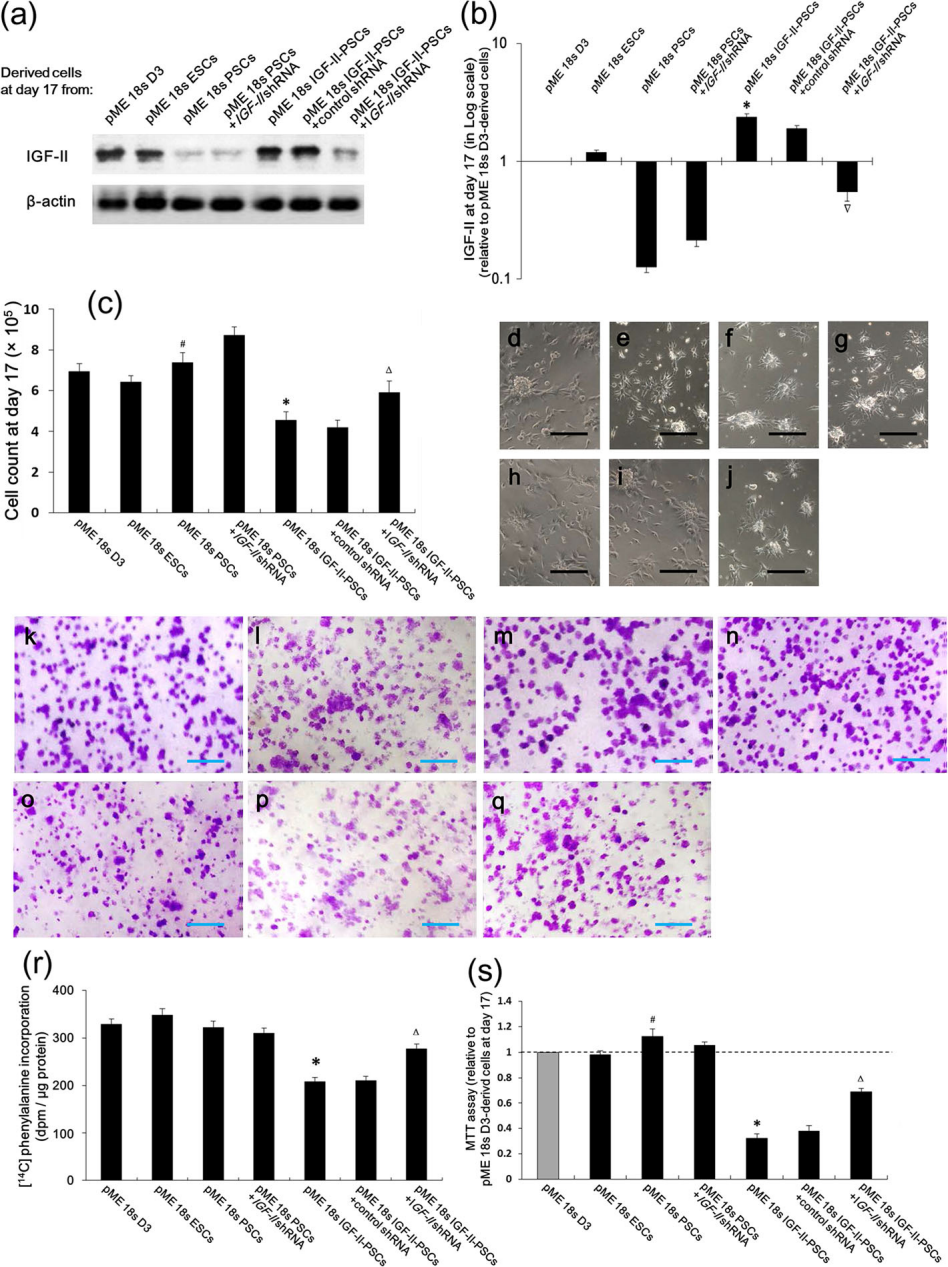
IGF-II overexpression inhibited PSC proliferation while promoting differentiation. pME 18s PSCs were stably transfected with IGF-II and/or shRNA to inhibit IGF-II, as indicated. **a** Western blot analysis was performed to detect IGF-II in cell derivatives from various clones at differentiation day 17. **b** Quantification of **a** (*n* = 6). IGF-II level was normalized to β-actin level and is presented in a logarithmic scale of the fold change relative to pME 18s D3-derived cardiomyocytes. **c** Cell numbers were counted at day 17 (*n* = 8). **d**–**j** Representative images of derivative live cultures at day 17 from pME 18s D3, pME 18s ESCs, pME 18s PSCs, pME 18s PSCs+IGF-II shRNA, pME 18s IGF-II-PSCs, pME 18s IGF-II-PSCs+control shRNA, and pME 18s IGF-II-PSCs+IGF-II shRNA, respectively. Scale bar, 50 μm. **k**–**q** Periodic acid-Schiff-stained images of cell derivatives of (**d**–**j**) on day 17. Scale bar, 1 mm. **r** [^14^C] phenylalanine incorporation analysis was performed to examine protein synthetic activity in cell derivatives from various derivatives on day 17 (*n* = 12). **s** MTT assay of cell derivatives on day 17 (*n* = 8). Data are presented as fold change relative to pME 18s D3-derived cells. *p < 0.05 vs. pME 18s PSCs; ∆*p* < 0.05 vs. pME 18s IGF-II-PSCs+control shRNA; #*p* < 0.05 vs. pME 18s D3 or pME 18s ESCs

To explore the suppressive effect of IGF-II overexpression on PSC proliferation, we re-knocked down IGF-II in IGF-II-PSCs (Fig. 5a, b). As shown, IGF-II re-knockdown rebooted the IGF-II-PSC count (Fig. 5j) that had been reduced by IGF-II overexpression (Fig. 5h). Consistent results were observed in periodic acid-Schiff (PAS) staining (Fig. 5k–q), [^14^C] phenylalanine incorporation (Fig. 5r), and MTT (Fig. 5s) assays.

### IGF-II overexpression activates the IGF1R/INSR signaling in PSCs

**Fig. 6:**
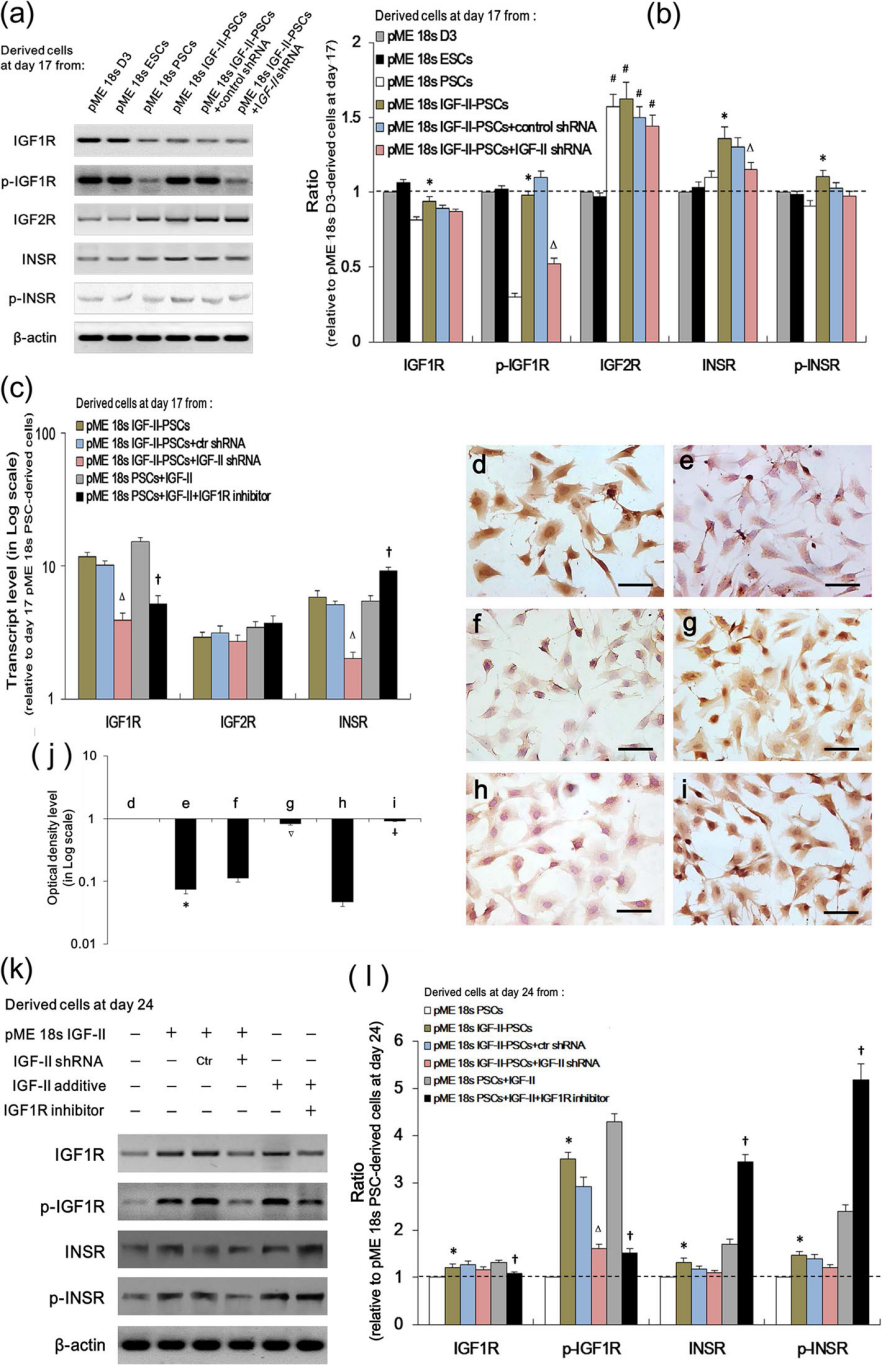
IGF-II overexpression activated the IGF1R/INSR signaling in PSCs. **a** Western blot analysis determined the IGF1R, p-IGF1R, IGF2R, p-IGF2R, INSR, and p-INSR protein levels in cell derivatives at differentiation day 17. **b** Quantification of **a**. Data are presented as the fold change relative to pME 18s D3-derived cells (*n* = 8). **c** qRT-PCR was performed to detect IGF1R, IGF2R, and INSR mRNA expression in cell derivatives on day 17 (*n* = 8). Data were normalized to β-actin and were presented on a logarithmic scale as the fold change relative to pME 18s PSC-derived cells. **d**–**i** Cyclin D2 (brown) immunocytochemical analysis at day 24 in cell derivatives from pME 18s PSCs (**d**), pME 18s IGF-II-PSCs (**e**), pME 18s IGF-II-PSCs+control (ctr) shRNA (**f**), pME 18s IGF-II-PSCs+IGF-II shRNA (**g**), pME 18s PSCs+IGF-II (**h**), and pME 18s PSCs+IGF-II+IGF1R inhibitor (**i**). Nuclei were counterstained with hematoxylin. Scale bar, 25 μm. **j** The average optical density (OD) of the stained cells in **d**–**i** was detected and was presented on a logarithmic scale as the fold change relative to pME 18s PSC-derived cells (*n* = 8). **k**, **l** Western blotting analysis for cell derivatives in different groups as indicated (**k**) and quantitative analysis (**l**). Band intensities in (**k**) were quantified, and the protein expression level was presented as the fold change relative to pME 18s PSC-derived cells (*n* = 8). *p < 0.05 vs. pME 18s PSCs; ∆*p* < 0.05 vs. pME 18s IGF-II-PSCs+control shRNA; †*p* < 0.05 vs. pME 18s PSCs+IGF-II

IGF-II signaling involves the receptors IGF-1R, IGF-2R, and INSR [21]. To explore which receptor mediates the differentiation-promoting and proliferation-inhibiting actions of IGF-II in PSCs, we detected these receptors’ protein expressions and phosphorylated (p-) active forms in PSCs with IGF-II manipulation. At differentiation day 17 (Fig. 6a, b), IGF-II overexpression elevated p-IGF1R expression in PSCs. This was counteracted by IGF-II shRNA. On the other hand, IGF2R expressions remained comparable in terms of PSCs, independent of IGF-II manipulation. Furthermore, IGF-II overexpression induced significant increases in INSR and p-INSR in PSCs compared with the empty vector. These data suggest that IGF1R and INSR, but not IGF2R, may be involved in IGF-II-regulated PSC differentiation.

Next, we detected the mRNA levels of these receptors in response to IGF-II treatment or IGF-II inhibitor. Adding IGF-II had the same inductive effects that IGF-II overexpression had on IGF1R and INSR mRNA expression in PSCs—both were significantly offset by IGF-II shRNA. This suggests that IGF-II is essential for IGF1R and INSR expression in PSCs (Fig. 6c). In addition, IGF1R inhibitor could significantly enhance INSR transcript expression, possibly due to regulatory compensation. In contrast, no significant changes were observed in IGF2R expression. Consistent results were observed in the protein expression of p-IGF1R and p-INSR at differentiation day 24 (Fig. 6k and i). Furthermore, immunocytochemical analysis of cyclin D2 at differentiation day 24 showed that both IGF-II shRNA (Fig. 6g) and IGF1R inhibitor (Fig. 6i) could significantly restore the cyclin D2 expression that had been suppressed by the overexpression (Fig. 6e) and addition (Fig. 6h) of IGF-II in PSCs. This suggests that IGF-II/IGF1R signaling plays an essential role in IGF-II-regulated PSC proliferation. Taken together, these findings suggest that IGF1R/INSR might mediate the effects of IGF-II on PSC proliferation.

### IGF-II overexpression promotes cardiomyocyte maturation in vivo

**Fig. 7:**
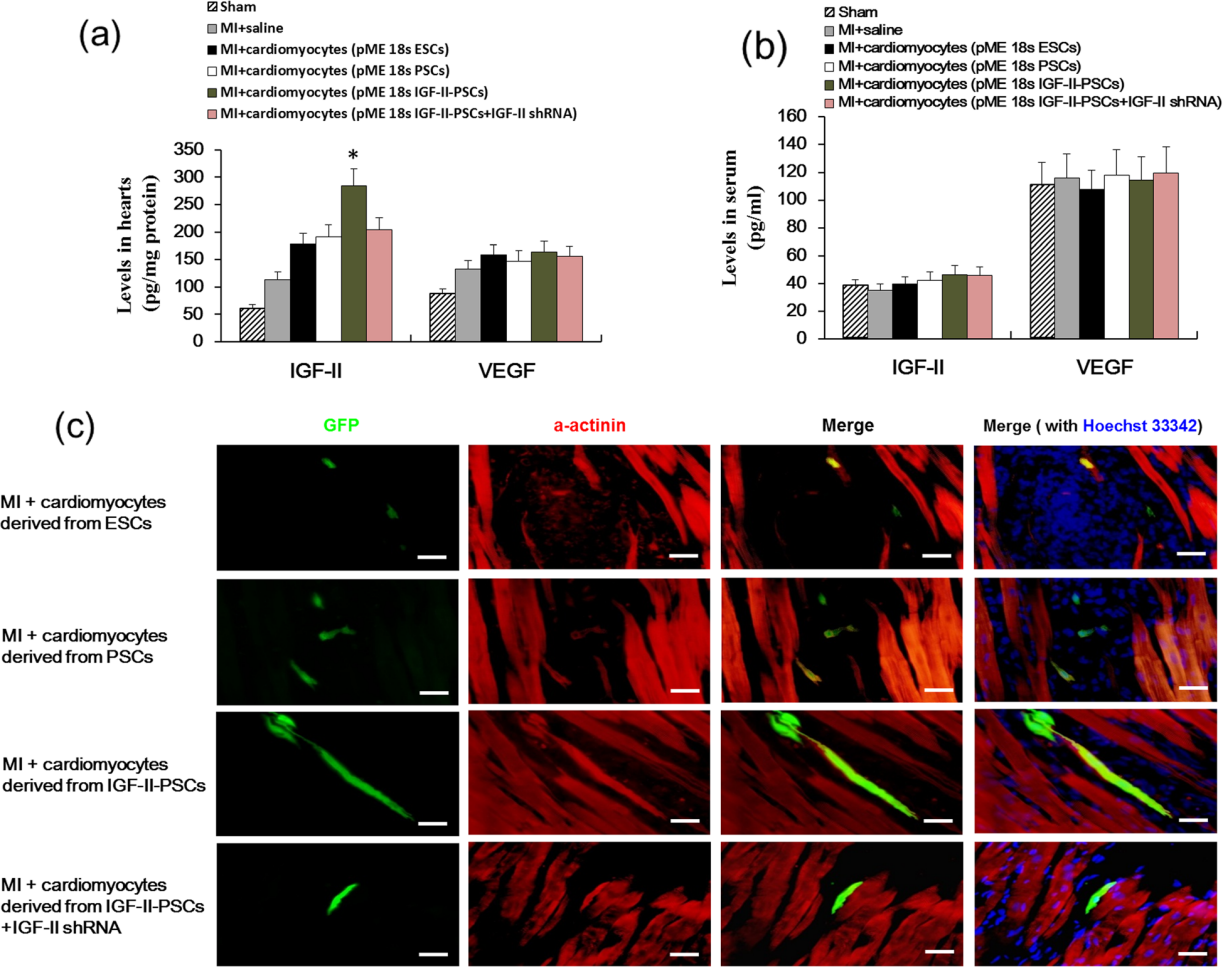
IGF-II overexpression promotes cardiomyocyte maturation in vivo. ESC- and PSC-derived cardiomyocytes at 1 × 10^5^ cells/recipient mouse were injected into the heart at 1 week after acute myocardial infarction (MI). The IGF-II and VEGF levels in the injection sites (**a**) and the serum (**b**) were measured by ELISA at 4 weeks post-transplant. Data are expressed as the mean ± SD. *p < 0.05 vs. any other group. *n* = 12. **c** Immunofluorescence staining was performed to observe the cardiomyocyte morphology and to detect α-actinin expression (red staining) in the injection sites at 4 weeks post-transplant. Blue staining, Hoechst 33342. Green staining, eGFP. Scale bars, 10 μm

To investigate the role of IGF-II in PSC differentiation and cardiomyocyte maturation in vivo, we re-knocked down IGF-II in IGF-II-overexpressing PSC clones. Knockdown efficiency was confirmed in the outgrowths by Western blot analysis (Fig. 5a, b). We then transplanted the derivatives of different clones into mice with acute MI. By 4 weeks post-transplant, although the retention rate of donor cardiomyocytes derived from IGF-II-PSCs remains modest at around 4.1%, IGF-II protein had significantly increased in the infarcted hearts (Fig. 7a), but not in the serum (Fig. 7b), of the mice that had received the IGF-II-PSC derivative transplants, compared with that of the other groups. This indicates the integration of the transplanted cells into the hearts.

We then evaluated the state of maturation in the cardiomyocytes derived from different clones based on cell morphology and sarcomeric α-actinin expression. As shown in Fig. 7c, IGF-II-PSC-derived cells displayed significant characteristics of mature cardiomyocytes, including increased elongation, distinct anisotropic rod shape, and enhanced α-actinin expression, compared with the control groups. IGF-II-depletion by shRNA significantly reversed these effects, suggesting the critical role of IGF-II in cardiomyocyte maturation in vivo. Moreover, IGF-II in vivo paracrine of donor cells derived from IGF-II-PSCs may have primarily accounted for the integration of transplanted donor cells into the host heart.

### IGF-II-PSC-derived cardiomyocytes improve pathologic changes and cardiac function post-MI

**Fig. 8:**
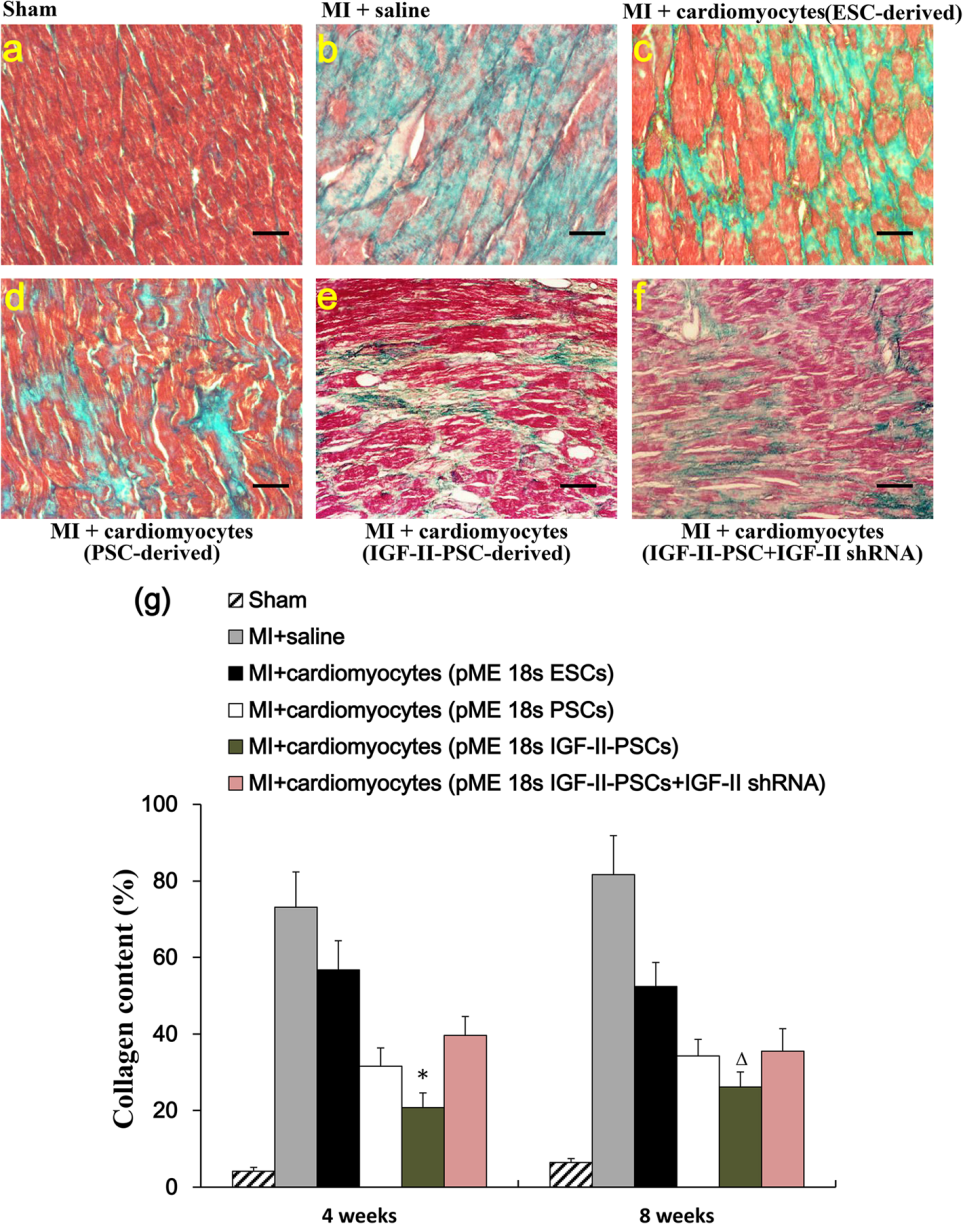
Collagen fiber detection by Masson’s trichrome staining. **a**–**f** At 4 weeks after transplant, Masson’s trichrome staining was performed to detect collagen fibrosis (blue staining) in the injection sites of mice in Sham (**a**), MI + saline (**b**), MI + cardiomyocytes derived from ESCs (**c**), MI + cardiomyocytes derived from PSCs (d), MI + cardiomyocytes derived from IGF-II-PSCs (**e**), and MI + cardiomyocytes derived from IGF-II-PSCs+IGF-II shRNA (**f**) groups, respectively. Representative images at 4 weeks post-transplant are shown. Scale bars, 30 μm. **g** Quantification of collagen content at 4 weeks and 8 weeks post-transplant, respectively. *p < 0.05 vs. any other group; Δ*p* < 0.05 vs. pME 18s IGF-II-PSCs+IGF-II shRNA group

**Fig. 9:**
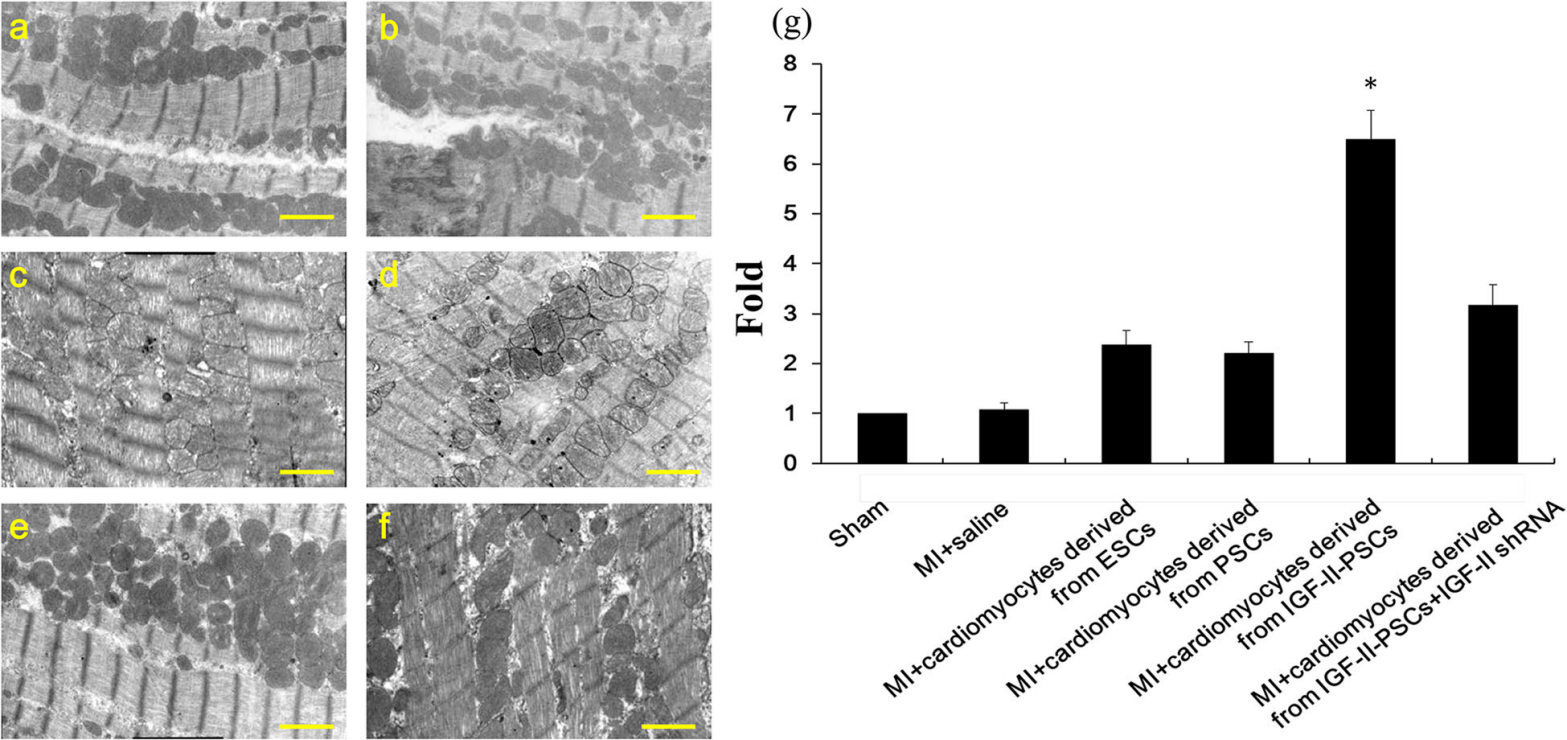
Cardiac mitochondrial morphology and density. **a**–**f** At 4 weeks after transplant, cardiac mitochondria were visualized under transmission electron microscopy in mice from Sham (**a**), MI + saline (**b**), MI + cardiomyocytes derived from ESCs (**c**), MI + cardiomyocytes derived from PSCs (**d**), MI + cardiomyocytes derived from IGF-II-PSCs (**e**), and MI + cardiomyocytes derived from the IGF-II-PSCs+IGF-II shRNA (**f**) groups, respectively. Representative images are shown. Scale bar, 20 nm. **g** Mitochondrion density quantification (normalized to Sham group). *p < 0.05 vs. any other group

**Fig. 10:**
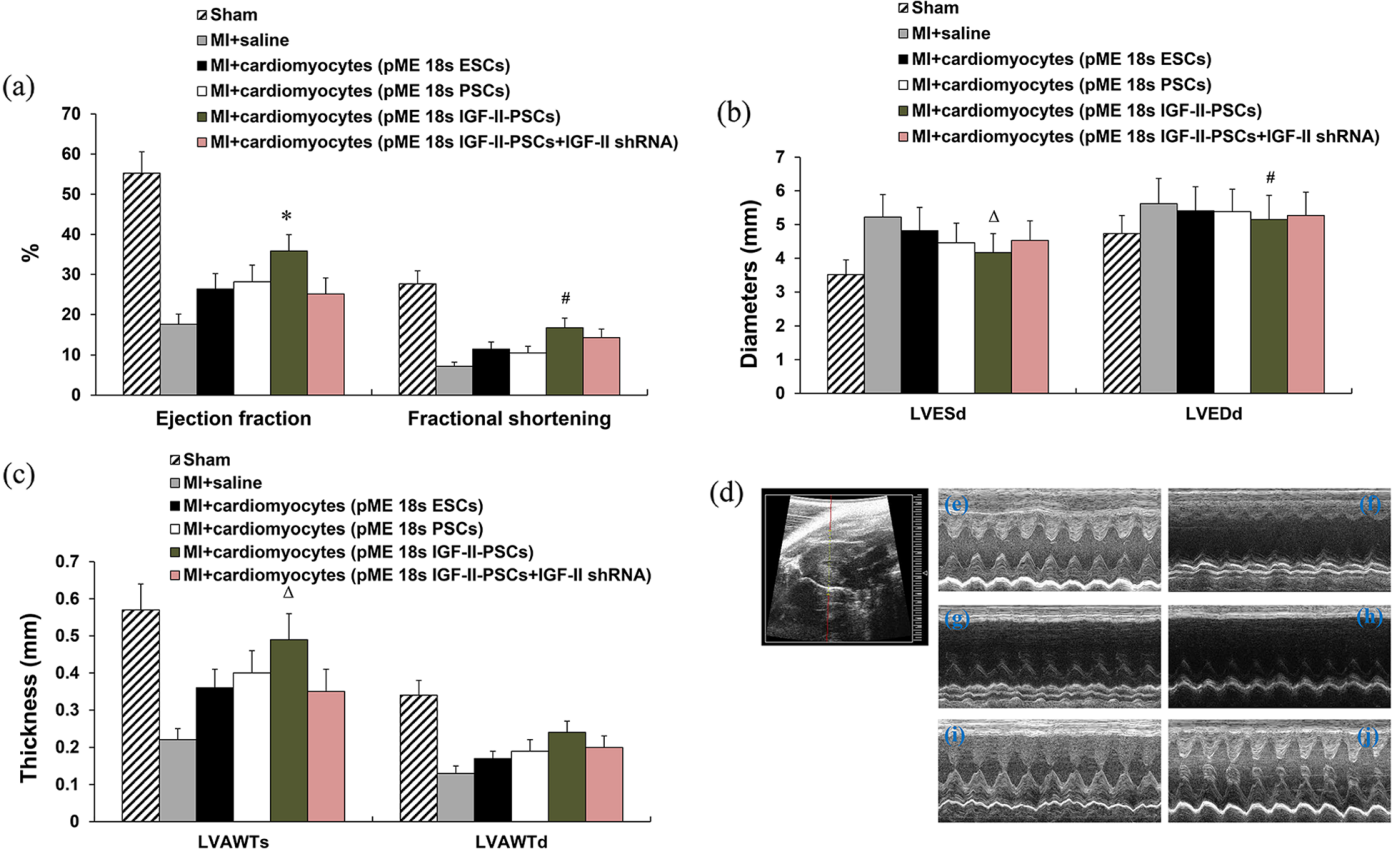
Cardiac function examination. At 4 weeks post-transplant (i.e., 5 weeks post-myocardial infarction, MI), ejection fraction (EF), fractional shortening (FS) (**a**), left ventricular end-systolic diameter (LVESd), left ventricular end-diastolic diameter (LVEDd) (**b**), left ventricular anterior wall thickness at end-systole (LVAWTs), and left ventricular anterior wall thickness at end-diastole (LVAWTd) (**c**) were determined to assess cardiac functions. *p < 0.05 vs. any other group; #*p* < 0.05 vs. pME 18s PSCs; Δ*p* < 0.05 vs. pME 18s IGF-II-PSCs+IGF-II shRNA; #*p* < 0.05 vs. pME 18s PSCs. **d**–**j** Representative short-axis echocardiograms, with heart positioning as indicated in **d**. **e** Sham. **f** MI + saline. **g** MI + cardiomyocytes (pME 18s ESCs). **h** MI + cardiomyocytes (pME 18s PSCs). **i** MI + cardiomyocytes (pME 18s IGF-II-PSCs). **j** MI + cardiomyocytes (pME 18s IGF-II-PSCs+IGF-II shRNA)

Next, we assessed the potential cardiac regenerative capacity of IGF-II-PSC-derived cardiomyocytes in MI by using Masson’s trichrome staining to detect collagen fibers (blue staining) at the injection sites. The results showed that IGF-II overexpression significantly reduced collagen deposits after MI, compared to the other groups. In contrast, IGF-II depletion negated this effect (Fig. 8a–g). Consistent results were observed for the density and morphology of mitochondria in the injection sites (Fig. 9a–g). Furthermore, transplant with IGF-II-PSC-derived cardiomyocytes improved indicators of cardiac function, such as ejection fraction, fractional shortening, left ventricular end-systolic diameter, left ventricular end-diastolic diameter, and left ventricular anterior wall thickness at end-systole, though not for left ventricular anterior wall thickness at end-diastole (Fig. 10a–c). Consistent results were observed in representative short-axis echocardiograms (Fig. 10d). Taken together, these results suggest that IGF-II-PSC-derived cardiomyocytes may reduce collagen deposition and promote mitochondria biogenesis, thereby improving cardiac function post-acute MI.

## Discussion

To date, with enormous progress in stem cell biology and technology, a decade-old enthusiasm with cell-based therapy and cardiac tissue engineering has not faded with the hope that progression of heart failure can be preempted. However, challenges remain in finding robust and stable sources of donor cardiomyocytes that can be immunologically tolerated by the recipient hearts. PSCs in mice have been found to exhibit similar fundamental properties to ESCs [10]. Previously, researchers presented a simple protocol of producing cardiomyocytes with easily generated IGF-II-overexpressing PSCs [10, 14] and showed that the engrafted PSC-derived cardiomyocytes could functionally integrate to the host myocardium [11]. This has made scalable production of donor cardiomyocytes readily accessible.

It has also been reported that transgenic overexpression of certain genes in mice can increase cardiomyocyte division [22]. Along these lines, genetic modification appears to be an attractive approach to optimize the regenerative potential of stem cells and break the barriers in the application of cell therapy in cardiomyocyte engraftment. Genetic modification has been used to stimulate stem cell differentiation and to designate new functions in cell reprogramming [23]. Much recent progress has been devoted to directing cell differentiation in adult stem cells, ESCs, and iPSCs [24–26].

Though an attractive candidate donor source of cardiomyocytes with haploidentity of major histocompatibility complex, a notable delay occurs in the differentiation of PSCs when compared to ESCs, which limits the therapeutic application of PSCs in cardiac engraftment [10, 11, 27]. A candidate gene that can alter this process is IGF-II, which is paternally imprinted and expected to be “erased” in PSCs but relatively overexpressed in ESCs [10, 12, 14].

Treatment with IGF-II was shown, during early differentiation (day 3), to direct murine ESCs towards a cardiac fate by promoting Brachyury^+^ mesodermal cell proliferation [28]. IGF-II-knockout ESCs exhibit severe impairment in cardiac and myogenic differentiation, which can be partially rescued by treatment with exogenous IGF-II [29]. In this study, we found that, compared with ESCs, IGF-II-deficient parental PSCs showed a delayed expression of cardiac markers (cTnT and cTnI) during differentiation, whereas forced expression of IGF-II erased this delay, supporting the role of IGF-II overexpression in promoting PSC differentiation into cardiomyocytes.

IGF-II can bind to an IGF1R/INSR hybrid or IGF2R and induce their phosphorylation, leading to cell proliferation and survival through mitogenic signaling [13]. Our results showed that IGF-II shRNA or IGF1R inhibitor can offset the IGF-II-induced activation of IGF1R and INSR, as well as restore IGF-II-suppressed cyclin D2 expression, arguing for an IGF1R/INSR-mediated mechanism. IGFR1 and INSR share 84% similarity in amino acid sequences and 100% in the ATP binding domain [30]. IGF1R and INSR can both bind with insulin, IGF-I, or IGF-II, as a monomer or hybrid polymer with differential affinities, but activate similar downstream effectors [31]. Blocking IGF1R activation may result in compensation through IGF signaling via INSR [32]. This might explain why IGF1R inhibitor could significantly enhance INSR mRNA expression in PSC derivatives.

Metabolically, the adult heart and the fetal heart are extremely different [33]. Moreover, immature mammalian hearts possess a robust capacity for cardiac regeneration that fades in adult hearts [34]. We found that IGF-II-PSC derivatives at differentiation day 24 expressed comparable amounts of cardiac markers to those of juvenile mouse cardiomyocytes, suggesting that IGF-II-PSCs at this stage may be an ideal source of cardiomyocyte for robust cardiac regeneration.

To improve cardiac myocyte maturation is one of the main goals of an effective therapeutic strategy. Immature cardiomyocytes are structurally and functionally different from mature ones [35]. Thus, the maturation state of newly differentiated cardiomyocytes is an important consideration. Although standardized metrics to determine cardiomyocyte maturity remain to be established, cell morphology is widely used as a convenient measurement [36]. Phenotypic properties of a mature human cardiomyocyte include highly organized sarcomeres, an anisotropic rod shape, and binucleation [37–39]. The sarcomere can be detected through the expression of α-actinin, cTnT, cTnI, and MHC isoforms [39, 40]. We found in this study that overexpression of IGF-II enables PSCs to differentiate towards mature cardiomyocytes. In addition, transplanted IGF-II-PSC-derivatives displayed highly organized sarcomere and enhanced α-actinin expression compared with the control groups, whereas IGF-II re-knockdown significantly reversed these effects. Together, these data suggest that IGF-II plays an essential role in promoting the maturation of PSC-derived cardiomyocytes in vivo.

Previously, we have demonstrated fundamental differences between murine PSCs and ESCs, with remarkable differences in allelic variability and differential imprinting characteristics of PSCs [10]. In particular, haploidentity of major histocompatibility complex in PSCs is suitable for allogeneic cell-based therapies, and we have verified tolerance of PSC-derived cell products in major histocompatibility complex-matched allotransplantation [10, 11]. The data presented in this study build on our previous research with murine PSCs, demonstrating genetic tractability of PSCs with IGF-II overexpression, and simplicity of generating pure populations of IGF-II-PSC-derived cardiomyocytes using a relatively scalable selection protocol. The beneficial effects of IGF-II in infarcted hearts have been previously reported in vivo. Exogenous IGF-II delivered to the infarcted area can improve myocardial function in swine by maintaining myocardial structure and inducing peri-infarct myocyte growth [41, 42]. Transplantation of IGF-II-overexpressing endothelial progenitor cells promotes cardiomyocyte proliferation while reducing the inflammatory response and apoptosis, thereby improving the left ventricular ejection fraction and reducing infarcted size in rats [43]. We also found a favorable effect of the cardiomyocytes derived from IGF-II-overexpressing PSCs in alleviating collagen deposition and improving mitochondria biogenesis and cardiac functions in the infarcted heart of mice. Removal of the IGF-II vector again nullified these effects, supporting its critical role in cardioprotection. In sum, IGF-II overexpression in PSCs promotes cardiomyogenic differentiation and maturation in vitro at the expense of proliferation, via activation of the IGF1R/INSR signaling, laying the foundation for integrating PSC-derived mature cardiomyocytes into host hearts after MI via IGF-II overexpression. After transplantation, our results suggest that PSC-derived cardiomyocytes with IGF-II overexpression may reduce collagen deposition and promote mitochondria biogenesis in the infarcted heart, thereby improving cardiac function post-acute MI.

## Conclusions

To the best of our knowledge, this was the first study to examine the effects of genetic modifications in promoting PSC differentiation towards cardiomyocytes. We demonstrated that the ectopic expression of IGF-II accelerates PSC differentiation into the cardiac lineage and promotes cardiomyocyte maturation. Also, the IGF-II/IGF1R signaling is involved in the suppressive effect of IGF-II on PSC proliferation. Furthermore, transplanting IGF-II-overexpressing PSC derivatives into the infarcted sites reduces collagen deposition and improves mitochondria biogenesis and cardiac function measurements. This suggests that IGF-II-overexpressing PSCs may be a promising source of cardiomyocytes in cardiac regenerative therapy.

## Data Availability

Data sharing is not applicable to this article as no datasets were generated or analyzed during the current study.

## Abbreviations

PSCs: Parthenogenetic stem cells
IGF-II: Insulin-like growth factor-II
MI: Myocardial infarction
ESCs: Human embryonic
iPSCs: Induced pluripotent stem cells
eGFP: Enhanced green fluorescence protein
DMRs: Differentially methylated regions
shRNA: Short hairpin RNA
LIF: Leukemia inhibitory factor
STO-CM: STO cell-conditioned medium
TRITC: Tetramethyl rhodamine isothiocyanate
PCNA: Proliferating cell nuclear antigen
PAS: Periodic acid-Schiff

## References

[1] Mohamed Tamer M.A., Ang Yen-Sin, Radzinsky Ethan, Zhou Ping, Huang Yu, Elfenbein Arye, Foley Amy, Magnitsky Sergey, Srivastava Deepak (2018). Regulation of Cell Cycle to Stimulate Adult Cardiomyocyte Proliferation and Cardiac Regeneration. Cell.

[2] Yu Jingyi, Seldin Marcus M., Fu Kai, Li Shen, Lam Larry, Wang Ping, Wang Yijie, Huang Dian, Nguyen Thang L., Wei Bowen, Kulkarni Rajan P., Di Carlo Dino, Teitell Michael, Pellegrini Matteo, Lusis Aldons J., Deb Arjun (2018). Topological Arrangement of Cardiac Fibroblasts Regulates Cellular Plasticity. Circulation Research.

[3] Cahill TJ, Kharbanda RK (2017). Heart failure after myocardial infarction in the era of primary percutaneous coronary intervention: mechanisms, incidence and identification of patients at risk. World J Cardiol.

[4] Bristow MR, Saxon LA, Boehmer J, Krueger S, Kass DA, De Marco T (2004). Cardiac-resynchronization therapy with or without an implantable defibrillator in advanced chronic heart failure. N Engl J Med.

[5] McMurray JJ, Packer M, Desai AS, Gong J, Lefkowitz MP, Rizkala AR (2014). Angiotensin-neprilysin inhibition versus enalapril in heart failure. N Engl J Med.

[6] Packer M, Coats AJ, Fowler MB, Katus HA, Krum H, Mohacsi P (2001). Effect of carvedilol on survival in severe chronic heart failure. N Engl J Med.

[7] Hashimoto H, Olson EN, Bassel-Duby R (2018). Therapeutic approaches for cardiac regeneration and repair. Nat Rev Cardiol.

[8] Fernandes S, Kuklok S, McGonigle J, Reinecke H, Murry CE (2012). Synthetic matrices to serve as niches for muscle cell transplantation. Cells Tissues Organs.

[9] Yamanaka S (2012). Induced pluripotent stem cells: past, present, and future. Cell Stem Cell.

[10] Didie M, Christalla P, Rubart M, Muppala V, Doker S, Unsold B (2013). Parthenogenetic stem cells for tissue-engineered heart repair. J Clin Invest.

[11] Yang T, Rubart M, Soonpaa MH, Didie M, Christalla P, Zimmermann WH (2015). Cardiac engraftment of genetically-selected parthenogenetic stem cell-derived cardiomyocytes. PLoS One.

[12] Nordin M, Bergman D, Halje M, Engstrom W, Ward A (2014). Epigenetic regulation of the Igf2/H19 gene cluster. Cell Prolif.

[13] Boone DN, Lee AV (2012). Targeting the insulin-like growth factor receptor: developing biomarkers from gene expression profiling. Crit Rev Oncog.

[14] Prelle K, Wobus AM, Krebs O, Blum WF, Wolf E (2000). Overexpression of insulin-like growth factor-II in mouse embryonic stem cells promotes myogenic differentiation. Biochem Biophys Res Commun.

[15] Sun Y, Gao D, Liu Y, Huang J, Lessnick S, Tanaka S (2006). IGF2 is critical for tumorigenesis by synovial sarcoma oncoprotein SYT-SSX1. Oncogene..

[16] Qin XF, An DS, Chen IS, Baltimore D (2003). Inhibiting HIV-1 infection in human T cells by lentiviral-mediated delivery of small interfering RNA against CCR5. Proc Natl Acad Sci U S A.

[17] He TC, Zhou S, da Costa LT, Yu J, Kinzler KW, Vogelstein B (1998). A simplified system for generating recombinant adenoviruses. Proc Natl Acad Sci U S A.

[18] Cury DP, Dias FJ, Sosthenes MC, Dos Santos Haemmerle CA, Ogawa K, Da Silva MC (2013). Morphometric, quantitative, and three-dimensional analysis of the heart muscle fibers of old rats: transmission electron microscopy and high-resolution scanning electron microscopy methods. Microsc Res Tech.

[19] Ruijtenberg S, van den Heuvel S (2016). Coordinating cell proliferation and differentiation: antagonism between cell cycle regulators and cell type-specific gene expression. Cell Cycle.

[20] Reddel RR (2014). Telomere maintenance mechanisms in cancer: clinical implications. Curr Pharm Des.

[21] He G, Kuang J, Koomen J, Kobayashi R, Khokhar AR, Siddik ZH (2013). Recruitment of trimeric proliferating cell nuclear antigen by G1-phase cyclin-dependent kinases following DNA damage with platinum-based antitumour agents. Br J Cancer.

[22] Zhu W, Reuter S, Field LJ (2019). Targeted expression of cyclin D2 ameliorates late stage anthracycline cardiotoxicity. Cardiovasc Res.

[23] Ziegler AN, Chidambaram S, Forbes BE, Wood TL, Levison SW (2014). Insulin-like growth factor-II (IGF-II) and IGF-II analogs with enhanced insulin receptor-a binding affinity promote neural stem cell expansion. J Biol Chem.

[24] Jung Y, Bauer G, Nolta JA (2012). Concise review: induced pluripotent stem cell-derived mesenchymal stem cells: progress toward safe clinical products. Stem Cells.

[25] Soto DA, Ross PJ (2016). Pluripotent stem cells and livestock genetic engineering. Transgenic Res.

[26] Yin PT, Han E, Lee KB (2016). Engineering stem cells for biomedical applications. Adv Healthc Mater.

[27] Verheyen A, Diels A, Reumers J, Van Hoorde K, Van den Wyngaert I, van Outryve d’Ydewalle C (2018). Genetically engineered iPSC-derived FTDP-17 MAPT neurons display mutation-specific neurodegenerative and neurodevelopmental phenotypes. Stem Cell Rep.

[28] Ho NTK, Nguyen TVT, Nguyen TV, Bui HT (2019). Epigenetic impairments in development of parthenogenetic preimplantation mouse embryos. J Reprod Dev.

[29] Engels MC, Rajarajan K, Feistritzer R, Sharma A, Nielsen UB, Schalij MJ (2014). Insulin-like growth factor promotes cardiac lineage induction in vitro by selective expansion of early mesoderm. Stem Cells.

[30] Porrello ER, Mahmoud AI, Simpson E, Hill JA, Richardson JA, Olson EN (2011). Transient regenerative potential of the neonatal mouse heart. Science..

[31] Chitnis MM, Yuen JS, Protheroe AS, Pollak M, Macaulay VM (2008). The type 1 insulin-like growth factor receptor pathway. Clin Cancer Res.

[32] Frasca F, Pandini G, Scalia P, Sciacca L, Mineo R, Costantino A (1999). Insulin receptor isoform A, a newly recognized, high-affinity insulin-like growth factor II receptor in fetal and cancer cells. Mol Cell Biol.

[33] Soonpaa MH, Zebrowski DC, Platt C, Rosenzweig A, Engel FB, Field LJ (2015). Cardiomyocyte cell-cycle activity during preadolescence. Cell..

[34] Morali OG, Jouneau A, McLaughlin KJ, Thiery JP, Larue L (2000). IGF-II promotes mesoderm formation. Dev Biol.

[35] Zhang H, Pelzer AM, Kiang DT, Yee D (2007). Down-regulation of type I insulin-like growth factor receptor increases sensitivity of breast cancer cells to insulin. Cancer Res.

[36] Davis RP, Casini S, van den Berg CW, Hoekstra M, Remme CA, Dambrot C (2012). Cardiomyocytes derived from pluripotent stem cells recapitulate electrophysiological characteristics of an overlap syndrome of cardiac sodium channel disease. Circulation..

[37] Besser RR, Ishahak M, Mayo V, Carbonero D, Claure I, Agarwal A (2018). Engineered microenvironments for maturation of stem cell derived cardiac myocytes. Theranostics..

[38] Pasqualini FS, Sheehy SP, Agarwal A, Aratyn-Schaus Y, Parker KK (2015). Structural phenotyping of stem cell-derived cardiomyocytes. Stem Cell Rep..

[39] Yang X, Pabon L, Murry CE (2014). Engineering adolescence: maturation of human pluripotent stem cell-derived cardiomyocytes. Circ Res.

[40] Robertson C, Tran DD, George SC (2013). Concise review: maturation phases of human pluripotent stem cell-derived cardiomyocytes. Stem Cells.

[41] Battler A, Hasdai D, Goldberg I, Ohad D, Di Segni E, Bor A (1995). Exogenous insulin-like growth factor II enhances post-infarction regional myocardial function in swine. Eur Heart J.

[42] McCain ML, Parker KK (2011). Mechanotransduction: the role of mechanical stress, myocyte shape, and cytoskeletal architecture on cardiac function. Pflugers Arch.

[43] Kotlyar AA, Vered Z, Goldberg I, Chouraqui P, Nas D, Fridman E (2001). Insulin-like growth factor I and II preserve myocardial structure in postinfarct swine. Heart..

